# Exercise for Girls

**Published:** 1891-10

**Authors:** 


### EXERCISE FOR GIRLS.

There is no greater fallacy current than the belief that the amount of exercise which should be beneficial is only bounded by the capacity of the person to take. Whether it be taken with a view to strengthen the muscles, or to invigorate the nervous system, exercise should always be gradual in its increase and accommodated to the actual state of the vital powers of the individual. We are not to consider that, because we were once capable of walking so many miles without fatigue, or performing some gymnastic feat, we are to try and keep up this power indefinitely, when the body has become less robust.

All that can be safely borne is regular and easy exercise of the body, continued over a long period, so as to give tone to the vital function without producing the exhaustion which inevitably follows upon any excessive demand upon their power.

There has been in some instances less headaches, in others marked improvement where various disturbances to health had existed. I look for benefit to all who practice regularly and faithfully. Gymnastics strengthen more sets of muscles than walking or rowing.

But regulated gymnastic exercise is only one means of physical culture ; modes of dress, out-of-door exercise, bathing and sleeping are equal in importance.

From neglect of precautions in childhood, which seem trifling, but are very important, there are few, if any, perfect forms. The shoulders are either too round, or one is higher than the other ; the neck is sunk too deep into the body or twisted ; the figure is thick, too thin or all of a piece, as it were, and the limbs are more or less distorted. When the shoulders of a young girl show a tendency to become too round, she must be made to throw her elbows well in the rear and her chest forward, and to sleep on her back. An hour's exercise every day under the eye of a judicious teacher of calisthenics is an excellent preventive of deformities.

The neck should be carried straight, but without stiffness ; in such a way, in fact, that the fleshy part below the jaw may form, as it were, a double chin.

If she will determine that as much as possible of the time of each day in which she is sitting down she will sit with her head and neck up, trunk erect and shoulders low, and that whenever she stands or walks she will at all times be upright, she will shortly find that she is getting to be far straighter than she was, and if she has a larger and finer chest than formerly it will be nothing strange, for she has simply been using one of the means to get it.

